# Molecular regulation of apoptotic machinery and lipid metabolism by mTORC1/mTORC2 dual inhibitors in preclinical models of HER2+/PIK3CAmut breast cancer

**DOI:** 10.18632/oncotarget.11490

**Published:** 2016-08-22

**Authors:** Jianchang Qian, Yaqing Chen, Tao Meng, Lanping Ma, Lanfang Meng, Xin Wang, Ting Yu, Arie Zask, Jingkang Shen, Ker Yu

**Affiliations:** ^1^ Department of Pharmacology, Fudan University School of Pharmacy, Shanghai, China; ^2^ Department of Medicinal Chemistry, State Key Laboratory of Drug Research, Shanghai Institute of Materia Medica, Chinese Academy of Sciences, Shanghai, China; ^3^ Department of Biological Sciences, Columbia University, New York, NY, USA

**Keywords:** mTOR kinase inhibitor, mTORC2, apoptosis, lipid metabolism

## Abstract

The mechanistic target of rapamycin (mTOR) is a rational target for cancer treatment. While the mTORC1-selective rapalogs have shown significant benefits in the clinic, antitumor response may be further improved by inhibiting both mTORC1 and mTORC2. Herein, we established target profile of a novel mTOR kinase inhibitor (mTOR-KI) MTI-31 and employed it to study new therapeutic mechanism in breast cancer. MTI-31 demonstrated a potent mTOR binding affinity with >5000 fold selectivity over the related PI3K family isoforms. MTI-31 inhibited mTORC1- and mTORC2 function at ≤120 nM in cellular assays or 5 mg/kg orally in tumor-bearing mice. In a panel of breast cancer lines, the antitumor efficacy of MTI-31 was dependent on HER2+ and/or PIK3CAmut (HER2+/PIK3CAmut) status of the tumors and required mTORC2-specific modulation of Bim, MCL-1 and GSK3. Inactivation of Bim or GSK3 each attenuated apoptotic death resulting in mTOR-KI resistance. The antitumor response also required a suppression of lipid metabolism in therapy-sensitive tumors. Treatment with MTI-31 or AZD8055 substantially reduced lipogenesis and acetyl-CoA homeostasis, which was mechanistically linked to a blockade of mTORC2-dependent glucose-to-lipid conversion rate. We also found that the basal levels of carnitine palmitoyltransferase 1A and lipid catabolism were elevated in HER2+/PIK3CAmut breast cells and were inhibited upon mTOR-KI treatment. A CPT1A inhibitor etomoxir mimicked MTI-31 action in selective downregulation of cellular lipid catabolism. Co-treatments with MTI-31 and etomoxir enhanced the suppression of cyclin D1, c-Myc and cell growth in HER2+/PIK3CAmut tumors. These new mechanistic findings provide a rationale for targeting mTORC1 and mTORC2 in HER2+/PIK3CAmut breast cancer.

## INTRODUCTION

The mechanistic target of rapamycin (mTOR) is a serine/threonine kinase related to the lipid kinases of the phosphoinositide 3-kinase (PI3K) family [1, 2]. mTOR is the catalytic subunit of two multiprotein complexes, mTOR complex-1 (mTORC1) and mTOR complex-2 (mTORC2), which differentially and coordinately function in parallel and downstream of the PI3K/AKT pathway. The PI3K/AKT/mTOR network is frequently dysregulated in tumorigenesis and represents a subject of intense discovery research [3–6]. The classical rapamycin-sensitive mTORC1 phosphorylates the ribosomal protein S6 kinase 1 (S6K1) and the eukaryotic translation initiation factor eIF4E-binding protein 1 (4EBP1) to promote mRNA translation and cell growth [3–7]. The more recently identified mTORC2 phosphorylates the survival kinase AKT and regulates cytoskeleton network [8, 9] with important implications in cancer survival, metabolism and tumor metastasis [8–14]. Clinically used rapamycin analogs, e.g. temsirolimus and everolimus, are allosteric inhibitors of mTORC1 [15, 16]. While these agents have achieved significant success in the clinic, antitumor response may be further improved by inactivation of both mTORC1 and mTORC2 [15–17]. ATP-competitive mTOR kinase inhibitors (mTOR-KIs) are capable of inhibiting both mTORC1 and mTORC2. To date, several such agents (e.g. AZD8055, INK/MLN-128, AZD2014 and CC-223) have entered patient trials with clinical results eagerly awaited [18–22]. In the preclinical setting, both rapalogs and mTOR-KIs are shown to inhibit cellular proliferation and attenuate process of aging [23–25]; Additional targeting of mTORC2-related survival function by the mTOR-KIs will offer deeper and broader antitumor activity. A major priority in developing new generation mTOR-targeted therapy is to elucidate molecular mechanism of mTOR complexes and to identify tumor subtype and underlying response biomarker that will help treatment stratification. Given that mTOR-KIs target both mTORC1 and mTORC2, characterization of mTORC-regulated cancer cell growth, survival and metabolism will provide new insight into targeted cancer treatment.

In this report, we first validated the biochemical and pharmacological profile of a novel mTOR-KI termed MTI-31 and established it as an excellent pharmacological tool for in vitro- and in vivo targeting of mTOR signaling pathways. Employing MTI-31 and earlier mTOR-KIs, we further demonstrated that the profound antitumor efficacy of the mTOR-KIs is dependent on cancer driver mutation status of the tumors and involves molecular regulation of apoptotic machinery and lipid metabolism in HER2+/PIK3CAmut breast cancer.

## RESULTS

### MTI-31 is a potent and highly selective inhibitor of mTORC1 and mTORC2

**Figure 1:**
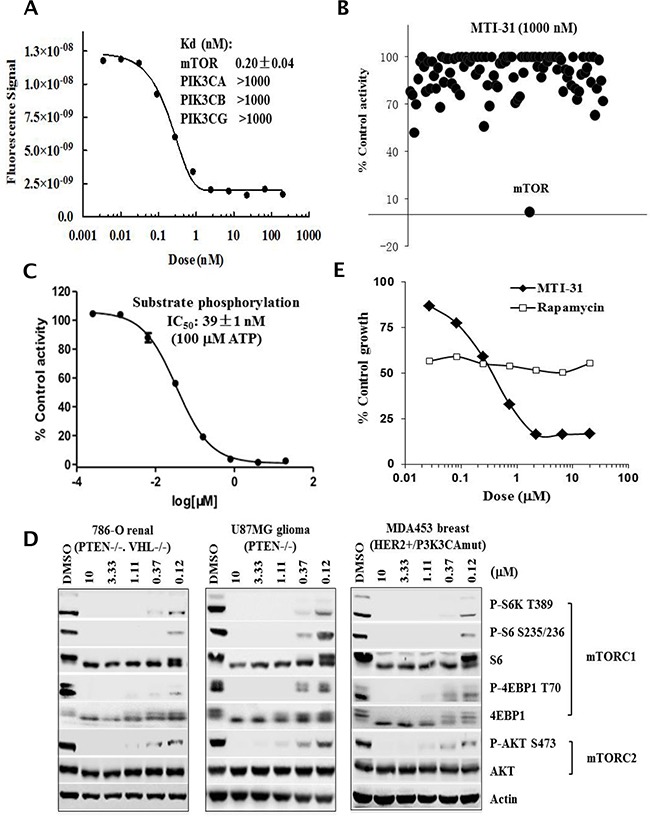
MTI-31 is a potent and selective mTOR kinase inhibitor **A.** Dose response curve of MTI-31 in mTOR binding assay; binding affinity for mTOR and related PI3K family members are shown. **B.** Binding activity profile of MTI-31 (1000 nM) in a panel of 98 kinases. The assays were performed via KINOMEscan as described in Methods. **C.** Dose response curve of MTI-31 in LANCE® assay measuring mTOR substrate phosphorylation as described in Methods. **D.** Cells of 786-O, U87MG and MDA-MB-453 were treated for 6 h with various doses of MTI-31 in growth medium and immunoblotted with the indicated mTORC1- and mTORC2 biomarkers. **E.** MDA-MB-453 cells were plated in 96-well plates, treated with various doses of rapamycin or MTI-31 for 3 days and assayed for cell proliferation.

Through efforts in inhibitor design, a series of new chemical inhibitors targeting both mTORC1 and mTORC2 have been synthesized [26]. To gain new insight into mTOR inactivation in cancer models, we characterized one such novel compound and designated it as mTOR inhibitor-31 (MTI-31). In mTOR binding assays, MTI-31 was potent and selective for mTOR (Kd: 0.20±0.04 nM) versus PIK3CA, PIK3CB and PIK3G with >5,000 fold selectivity (Figure 1A). Similar assays against 98 human kinases using a fixed concentration of MTI-31 (1000 nM) yielded mostly no or weak binding (Figure 1B, Supplementary Table S1). In LANCE® assay of mTOR substrate phosphorylation with 100 μM ATP, MTI-31 showed an IC_50_ value 39±1 nM (Figure 1C). In three representative tumor cell lines harboring mTOR pathway dysregulation (786-O renal, U87MG glioma and MDA-MB-453 breast), treatment with MTI-31 for 6 h demonstrated a dose-dependent inhibition of both the mTORC1 substrates P-S6K1(T389), P-S6(S235/6), P-4EBP1(T70) and mTORC2 substrate P-AKT(S473), achieving 50% inhibition at ≤0.12 μM (Figure 1D). Growth inhibition assay showed that compared to rapamycin, MTI-31 elicited a potent and more substantial inhibition of cell growth than that of rapamycin. The nM IC_50_ of MTI-31 in growth inhibition correlated well with its target potency in suppression of mTOR signaling biomarkers (Figure 1D, 1E). Together, these results have established MTI-31 as a potent and selective inhibitor of mTOR enzymatic activity capable of targeting both mTORC1 and mTORC2 functions in cancer cells.

### MTI-31 is a potent mTOR inhibitor in vivo and elicits strong antitumor efficacy

**Figure 2:**
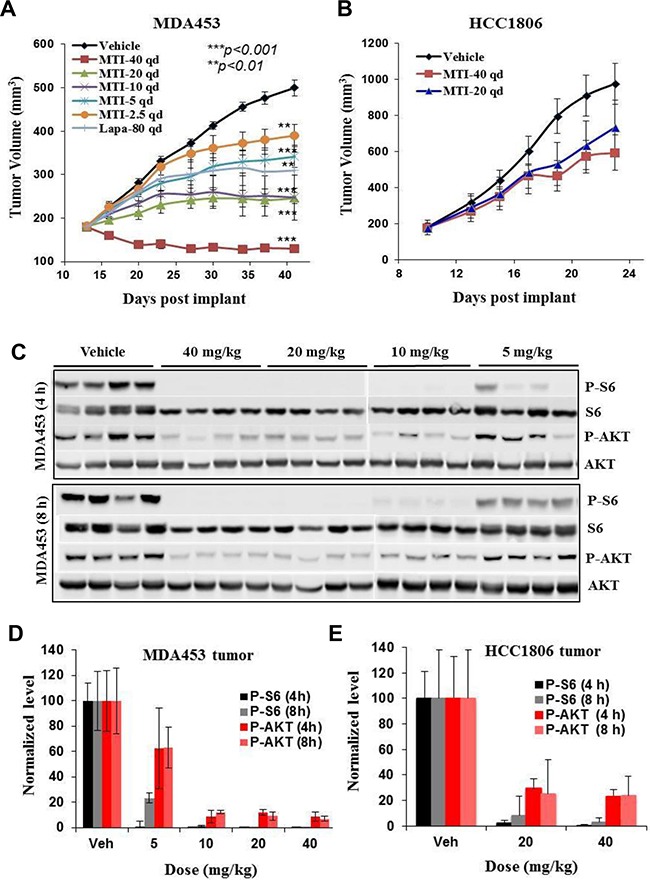
In vivo antitumor efficacy and mTOR-biomarker suppression profile of MTI-31 **A** and **B.** Female nude mice bearing tumors of MDA-MB-453 (A) or HCC1806 (B) were treated orally with various doses of MTI-31 or Lapatinib via a once daily (qd) regimen. Tumor growth curves are shown. **C.** On final day of study, following the last administration of MTI-31, tumors were collected and immunoblotted. **D** and **E.** Immunoblotting results are quantified and plotted for tumors of MDA-MB-453 (D) and HCC1806 (E). ***, P < 0.001; **, P < 0.01.

MTI-31 was efficacious in several tumor models harboring HER2+/PIK3CAmut and/or PTEN-deficiency exemplified by MDA-MB-453 (Figure 2A) and 786-O (Supplementary Figure S1A). In these models, orally administrated MTI-31 demonstrated a dose proportional tumor growth inhibition (TGI) with a minimum efficacious dose (MED) of 5 mg/kg (>50% TGI, p<0.01) and a maximum tolerated dose (MTD) of 40 mg/kg (7-15% body weight loss without mortality). In contrast, MTI-31 had limited efficacy in the HER2-/PIK3CAwt HCC1806 breast tumor model even at the highest 40 mg/kg (Figure 2B). The antitumor activity of MTI-31 in MDA-MB-453 tumors correlated well with its inhibition of mTORC1 and mTORC2 biomarkers in the tumor tissues as shown by a significantly reduced P-S6 and P-AKT levels at 5 mg/kg and a nearly complete suppression of both biomarkers at 10 mg/kg (Figure 2C, 2D). The TGI of MTI-31 also correlated well with its plasma exposure level measured as area under curve (AUC) or maximum concentration (Cmax) (Supplementary Figure S1B). Notably, a similar reduction in mTOR biomarker in HCC1806 tumors by 20 or 40 mg/kg MTI-31 did not lead to a strong antitumor efficacy (Figure 2B, 2E, Supplementary Figure S1C). These results indicate that MTI-31 is a potent inhibitor of mTORC1/mTORC2 signaling in vivo and a promising antitumor agent in the HER2/PIK3CA/mTOR-hyperactive tumor model.

### Breast cancer cell suppression profile by MTI-31 is dependent on cancer driver mutation status

**Figure 3:**
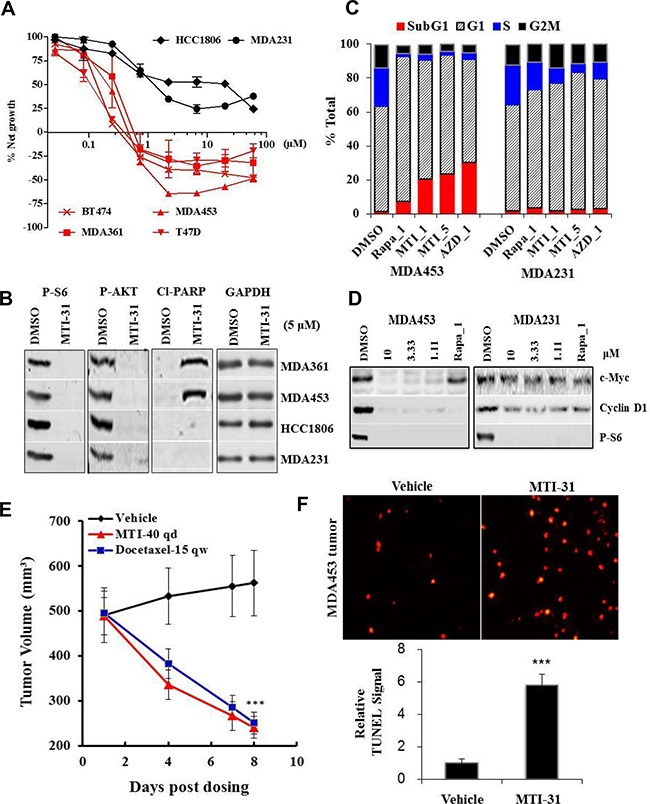
MTI-31 antitumor activity correlates with cancer driver mutations **A.** The indicated 6 breast cancer cell lines were treated with various doses of MTI-31 for 3 days, analyzed for net cell growth and death and survival dose response curves are plotted. **B.** The indicated 4 cell lines were treated with MTI-31 for 48 h and immunoblotted. **C.** Cell cycle profile of MDA-MB-453 and MDA-MB-231 cells after 48 h treatment with inhibitors as analyzed by FACS. Percentages of total cells in each cell cycle stage are shown. **D.** MDA-MB-453 and MDA-MB-231 cells were treated with the indicated inhibitors for 6 h and immunoblotted. **E.** Nude mice bearing large size MDA-MB-453 tumors were treated with 40 mg/kg MTI-31 qd or 15 mg/kg docetaxel qw. Tumor regression curves are shown. **F.** MDA-MB-453 tumors from vehicle- and 40 mg/kg MTI-31-treated mice were subjected to TUNEL assay as described in Methods. TUNEL images (n=3) were acquired and quantified. ***, P < 0.001; **, P < 0.01.

We next performed cell growth and survival assays using MTI-31 and AZD8055 in a panel of 6 breast tumor cell lines. The panel included the lines with HER2+ and/or PIK3CAmut (HER2+/PIK3CAmut) status (MDA-MB-453, MDA-MB-361, BT474 and T47D) or those with HER2-/PIK3CAwt status (MDA-MB-231 and HCC1806). After treatment for 3 days, MTI-31 was significantly more potent in growth inhibition and inducing cell death in all four HER2+/PIK3CAmut lines while it did not cause cell death in the two HER2-/PIK3CAwt lines even at high concentrations (Figure 3A). Accordingly, MTI-31 induced a strong increase in cleaved-PARP in MDA-MB-453 and MDA-MB-361 cells but not in MDA-MB-231 and HCC1806 cells, indicating a differential induction of apoptosis between the two cell types (Figure 3B). The assay with AZD8055 yielded a similar selective killing of the HER2+/PIK3CAmut cells (Supplementary Figure S2A). Cell cycle analysis with MDA-MB-453 and MDA-MB-231 cells showed that while rapamycin and mTOR-KIs MTI-31 and AZD8055 all increased G_1_ phase arrest in both cell lines, the decline in S phase and induction of cell death (Sub-G_1_) were both highly pronounced in the MTI-31- and AZD8055-treated MDA-MB-453 cells (Figure 3C). The differential loss of viability in MDA-MB-453 cells correlated with a nearly complete suppression of cyclin D1 and c-Myc as well as the activation of apoptosis machinery in MDA-MB-453 cells but not in MDA-MB-231 cells (Fig. 3D, Supplementary Figure S2B). In nude mice bearing large size MDA-MB-453 tumors, treatment with 40 mg/kg MTI-31 resulted in a rapid and dramatic tumor regression (Figure 3E). TUNEL staining showed that there was a significant increase in apoptosis in the treated MDA-MB-453 tumor tissue (p<0.001) (Figure 3F). Collectively, these results demonstrated that the HER2+/PIK3CAmut breast cancer cells are highly susceptible to mTOR-targeted growth suppression, which is mediated through cell cycle blockade and induction of apoptotic cell death.

### MTI-31-induced apoptosis requires mTORC2-regulated Bim- and GSK3 activity

**Figure 4:**
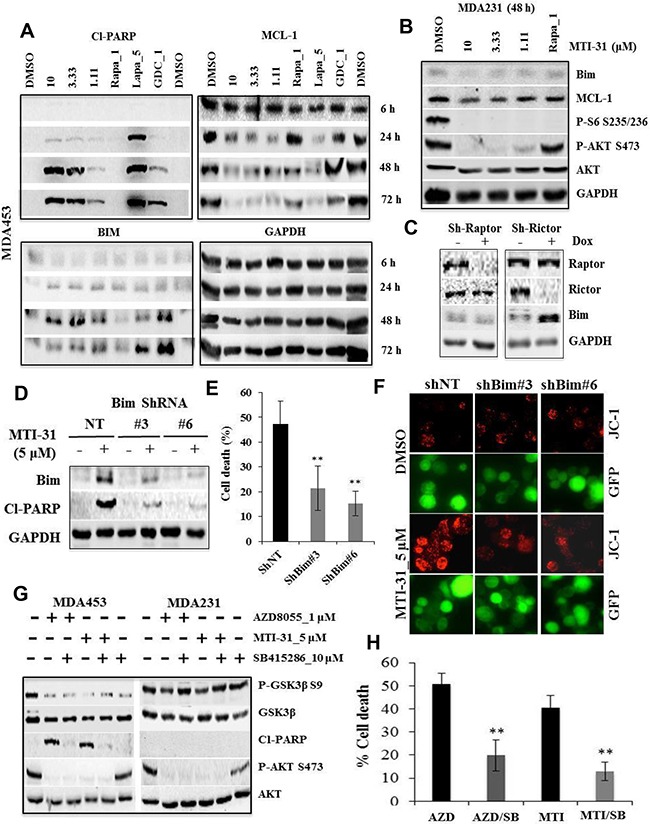
The MTI-31-induced apoptosis requires Bim- and GSK3 activity **A** and **B.** Cells of MDA-MB-453 (A) or MDA-MB-231 (B) were treated as indicated. Total cell lysates were immunoblotted. **C.** MDA-MB-453 cells stably infected with doxycycline (Dox)-inducible Raptor-ShRNA#2 or Rictor-ShRNA#4 were induced with Dox for 7 days and immunoblotted. **D-F.** MDA-MB-453 cells were infected with GIPZ-lentivirus encoding NT-ShRNA, Bim-ShRNA#3 and Bim-ShRNA#6. Puromycin-selected cells plated in parallel sets were treated for 48 h with MTI-31, subjected to immunoblotting (D) or survival measurement via counting live GFP-positive cells (E), or cell treatment for 18 h prior to JC-1 staining. JC-1 fluorescence images are shown (F). **G** and **H.** MDA-MB-453 and MDA-MB-231 cells were treated as indicated, subjected to immunoblotting 48 h later (G) and cell survival measurement 72 h later via cell counting; mean values of cell death (%) are plotted (H). **, P < 0.01.

Employing MDA-MB-453 as a representative HER2+/PIK3CAmut breast tumor model, we investigated molecular mechanism of apoptosis induced by MTI-31. Cells were treated with various doses of MTI-31, 1 μM rapamycin, 5 μM Lapatinib (HER2 inhibitor) and 1 μM GDC-0941 (PI3K inhibitor) followed by immunoblotting at 6, 24, 48 and 72 h later. MTI-31 induced a dose-dependent PARP-cleavage peaking at 48 h. The onset of apoptosis occurred following an induction of Bim and downregulation of MCL-1, indicating a regulatory control of apoptotic machinery by mTOR in these cells (Figure 4A). A similar induction time course for Bim was observed in AZD8055-treated MDA-MB-453 cells (Supplementary Figure S2C). Notably, 1 μM rapamycin caused only modest changes in Bim or MCL-1 expression consistent with a minor or no increase in cleaved-PARP (Figure 4A). Importantly, treatment of MDA-MB-231 cells with MTI-31 for 48 h failed to induce Bim or inhibit MCL-1 even though it efficiently suppressed both the mTORC1 and mTORC2 signaling functions (Figure 4B). Because mTORC2 is largely resistant to rapamycin, we investigated the relative contribution of the mTOR complexes to Bim induction. Depletion of rictor (disrupting mTORC2) but not raptor (disrupting mTORC1) resulted in a significant accumulation of Bim indicating that Bim induction involves largely the targeting of mTORC2 (Figure 4C). We further investigated the functional importance of Bim in mediating MTI-31-induced apoptosis. Depletion of Bim via two independent ShRNAs (ShBim#3 and ShBim#6) each blocked the MTI-31-induced Bim accumulation and cleaved-PARP and attenuated apoptosis (p<0.01) (Figure 4D, 4E). Likewise, depletion of Bim also attenuated the apoptosis-associated mitochondrial hyperpolarization in response to MTI-31 treatment (Figure 4F). Separately, we investigated whether glycogen synthase kinase 3 (GSK3), a rapamycin-resistant mTORC2 pathway target, also played a role in apoptosis. Treatment with MTI-31 or AZD8055 induced GSK3β Ser-9 dephosphorylation (activation) in the MDA-MB-453 but not MDA-MB-231 cells (Figure 4G). Importantly, inhibition of GSK3 enzymatic activity with a GSK3 kinase inhibitor SB415286 significantly reduced the MTI-31- or AZD8055-induced PARP-cleavage and cell death (p<0.01) (Figure 4G, 4H). Together, these results highlight an important mechanistic role for the rapamycin-resistant mTORC2 in survival of HER2/PIK3CA-hyperactive breast cancer cells via regulation of Bim and GSK3 activity.

### Inhibition of acetyl-CoA and de novo lipid synthesis is associated with antitumor efficacy

**Figure 5:**
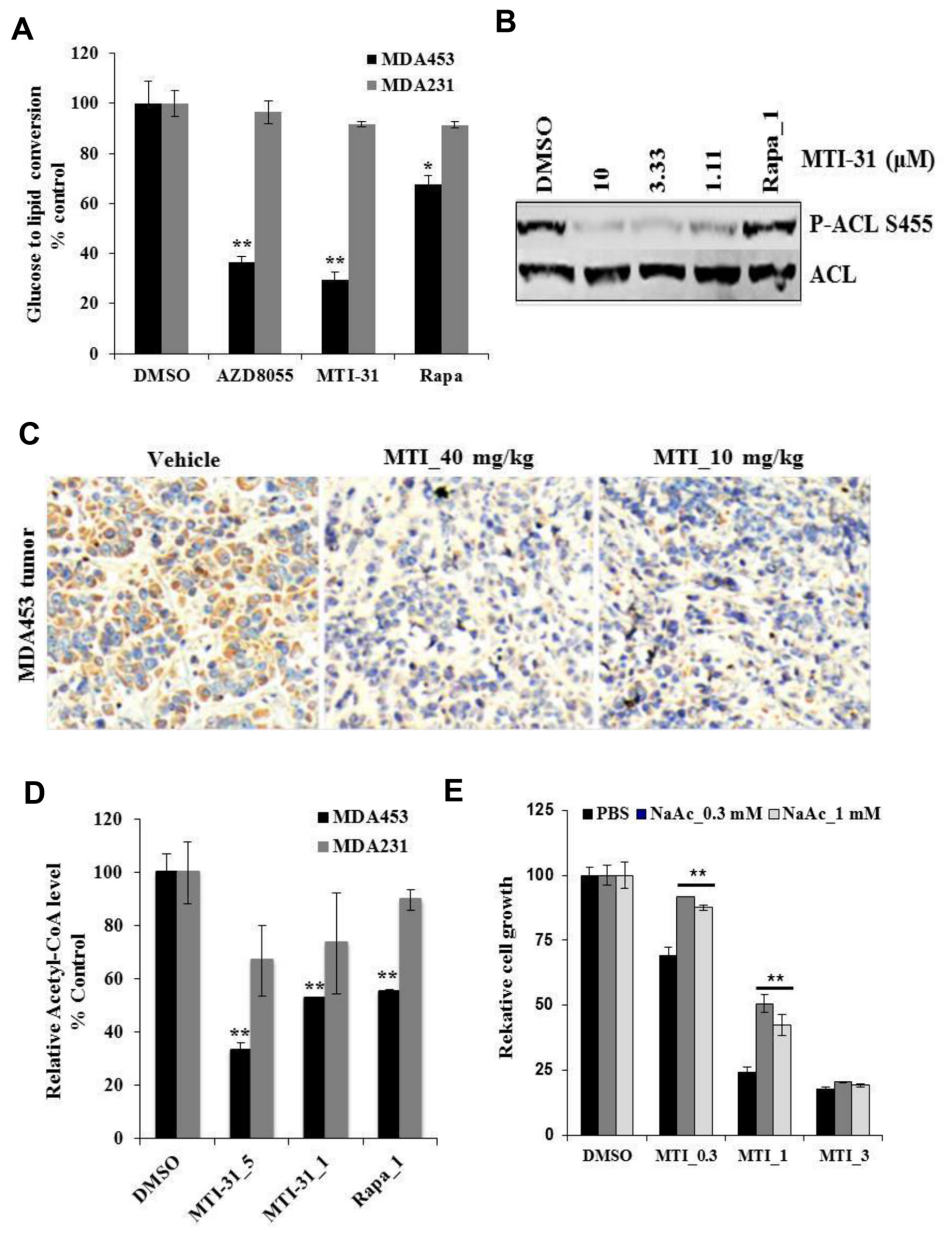
MTI-31 inhibits acetyl-CoA and de novo lipid synthesis **A.** MDA-MB-453 and MDA-MB-231 cells were grown in 12-well culture plates and treated with DMSO, 5 μM MTI-31, 1 μM rapamycin for 2 h, then assayed for glucose-to-lipid conversion as described in Methods. Normalized values are plotted. **B.** MDA-MB-453 cells were treated as indicated for 6 h and immunoblotted. **C.** MDA-MB-453 tumors from vehicle-, 40 and 10 mg/kg MTI-31-treated mice were subjected to IHC analysis with an anti-P-ACL antibody as described in Methods. **D.** MDA-MB-453 and MDA-MB-231cells were treated with DMSO, 5 and 1 μM MTI-31, 1 μM rapamycin for 24 h and assayed for total cellular acetyl-CoA level as described in Methods. Normalized values are plotted. **E.** MDA-MB-453 cells were treated with the indicated doses of MTI-31 without or with 0.3 or 1 mM NaAc for 4 days. Normalized cell growth values are plotted. **, P < 0.01; *, P < 0.05.

While it is increasingly recognized that de novo lipid synthesis is an important aspect of cancer metabolism, elucidating the role and mechanism of mTOR pathway in this process, particularly in breast cancer molecular subtype, may aid therapeutic strategy. We found that treatment of MDA-MB-453 cells for 4 h with a saturating dose of MTI-31, AZD8055 or rapamycin each significantly reduced cellular de novo lipid synthesis measured as a reduced ^14^C-glucose-to-lipid conversion rate (Figure 5A); Notably, both MTI-31 and AZD8055 caused a significantly stronger suppression of lipid synthesis compared to that of rapamycin (70% and 63% versus 32%, respectively) (Figure 5A). Importantly, similar treatment of MDA-MB-231 cells did not significantly reduce lipid synthesis (Figure 5A). We then examined effect of MTI-31 on ATP citrate lyase (ACL), an enzyme that converts citrate to lipogenic precursor acetyl-CoA. Immunoblotting and immunohistochemistry (IHC) showed that ACL Ser-455 phosphorylation in MDA-MB-453 was inhibited by MTI-31 in cultured cells (Figure 5B) and in tumor-bearing mice (Figure 5C) but was not inhibited by rapamycin (Figure 5B). Consistent with the differential loss of ACL phosphorylation, there was a greater reduction in acetyl-CoA in cells treated with MTI-31 (67%) than that of rapamycin (45%) (Figure 5D). Notably, the inhibition of acetyl-CoA by MTI-31 was significantly less in the identically treated MDA-MB-231 cells suggesting a differential dependence of mTOR pathway in acetyl-CoA homeostasis in these two cell types (Figure 5D). We then assayed MDA-MB-453 response to MTI-31 without or with an alternate source of acetyl-CoA via sodium acetate (NaAc). Interestingly, cells supplemented with 0.3 mM or 1 mM NaAc both demonstrated a partial but significant rescue from the growth suppression effects by 0.3 μM or 1 μM MTI-31 (p<0.01) (Figure 5E). Collectively, these results reveal an essential role for mTOR in de novo lipogenic process particularly in HER2/PIK3CA-hyperactive breast cancer cells like MDA-MB-453.

### Down-regulation of CPT1A and lipid catabolism plays a role in antitumor efficacy

**Figure 6:**
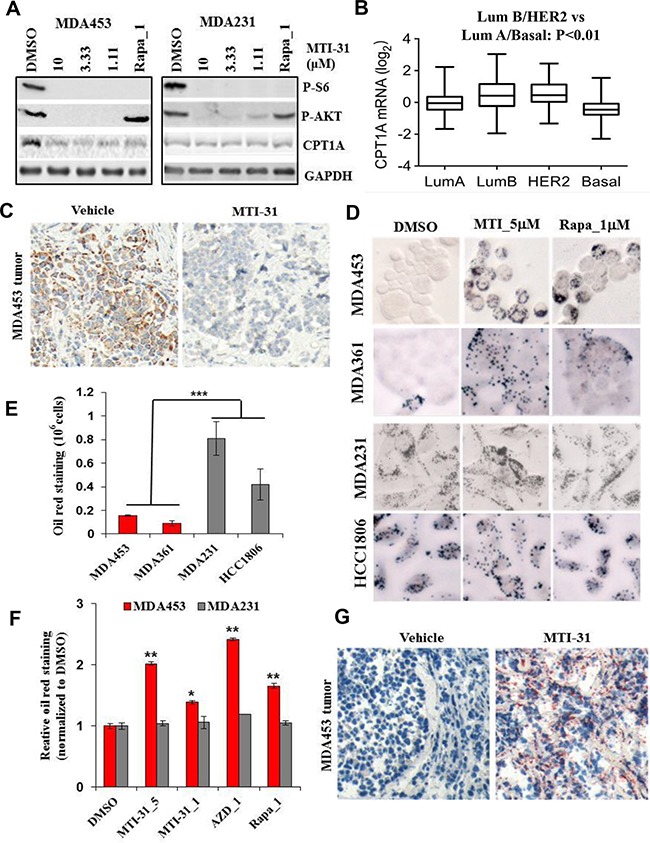
MTI-31 inhibits lipid catabolism **A.** MDA-MB-453 and MDA-MB-231 cells were treated as indicated for 24 h and immunoblotted. **B.** CPT1A mRNA expression levels as analyzed from The Cancer Genome Atlas (TCGA) research network (http://cancergenome.nih.gov). The 457 invasive breast carcinoma tissue sample data set was divided into 4 groups as defined by Prediction analysis of microarrays PAM50 [51]. The scatter plot was conducted by using software Prism 6.0 with corresponding value of each group. **C.** MDA-MB-453 tumors from vehicle- and 40 mg/kg MTI-31-treated mice were subjected to IHC analysis with an anti-CPT1A antibody as described in Methods. **D-F.** MDA-MB-453, MDA-MB-361, MDA-MB-231 and HCC1806 cells were treated with DMSO, 5 and 1 μM MTI-31, 1 μM AZD8055, 1 μM rapamycin for 24 h and stained with Oil red O as described in Methods. Images were acquired (D) and quantified via absorbance of dye-elution. Cell number-normalized staining signal in basal untreated cells (E) and inhibitor-treated cells (F) are shown. **G.** MDA-MB-453 tumors from vehicle- and 40 mg/kg MTI-31-treated mice were subjected to Oil red O staining analysis as described in Methods. ***, P < 0.001; **, P < 0.01.

As lipid synthesis and utilization could be complementary in supporting cancer cell growth, we next examined effect of mTOR-inhibition on lipid catabolism. We found that the basal protein expression of carnitine palmitoyltransferase 1A (CPT1A), a rate-limiting enzyme for fatty acid β-oxidation (FAO), was higher in the HER2+/PIK3CAmut MDA-MB-453 cells compared to MDA-MB-231 cells (Figure 6A, Supplementary Figure S3A). Analysis of The Cancer Genome Atlas (TCGA) databank showed that CPT1A mRNA level is significantly higher in Luminal B and HER2+ breast tumors (Figure 6B). A similar observation was made in analysis of Cancer Cell Line Encyclopedia (CCLE) databank (not shown). Treatment with MTI-31, AZD8055 or rapamycin each significantly reduced CPT1A protein expression in MDA-MB-453 cells but the inhibition was not evident in MDA-MB-231 cells (Figure 6A, Supplementary Figure S3A). The CPT1A inhibition was time dependent and also sensitive to HER2 or PI3K inhibitors (Supplementary Figure S3B). IHC analysis showed a substantial reduction in CPT1A protein level in MDA-MB-453 tumors in mice treated with 40 mg/kg and 10 mg/kg MTI-31 (Figure 6C, Supplementary Figure S3C). We then performed Oil-red O staining to measure the cellular lipid levels, which may change in response to a reduced lipid catabolism. The basal lipid droplet level was low in MDA-MB-453 and MDA-MB-361 cells and was greatly increased upon 24 h treatment with MTI-31, AZD8055 or rapamycin. In contrast, the basal lipid droplet level was higher in MDA-MB-231 and HCC1806 cells and was largely unchanged upon mTOR inhibition (Figure 6D, 6E, 6F). Consistent with the results from cultured cells, there was a dramatic increase in Oil red O staining in the MDA-MB-453 tumors treated with 40 mg/kg and 10 mg/kg MTI-31 (Figure 6G, Supplementary Figure S3D). These results collectively suggest that the HER2+/PIK3CAmut breast tumor cells are associated with a state of elevated CPT1A activity and a robust lipid utilization, both of which are connected to mTOR and are inhibited by mTOR-KIs or rapamycin.

### CPT1A inhibitor selectively blocks lipid catabolism and growth in MTI-31-sensitive cells

**Figure 7:**
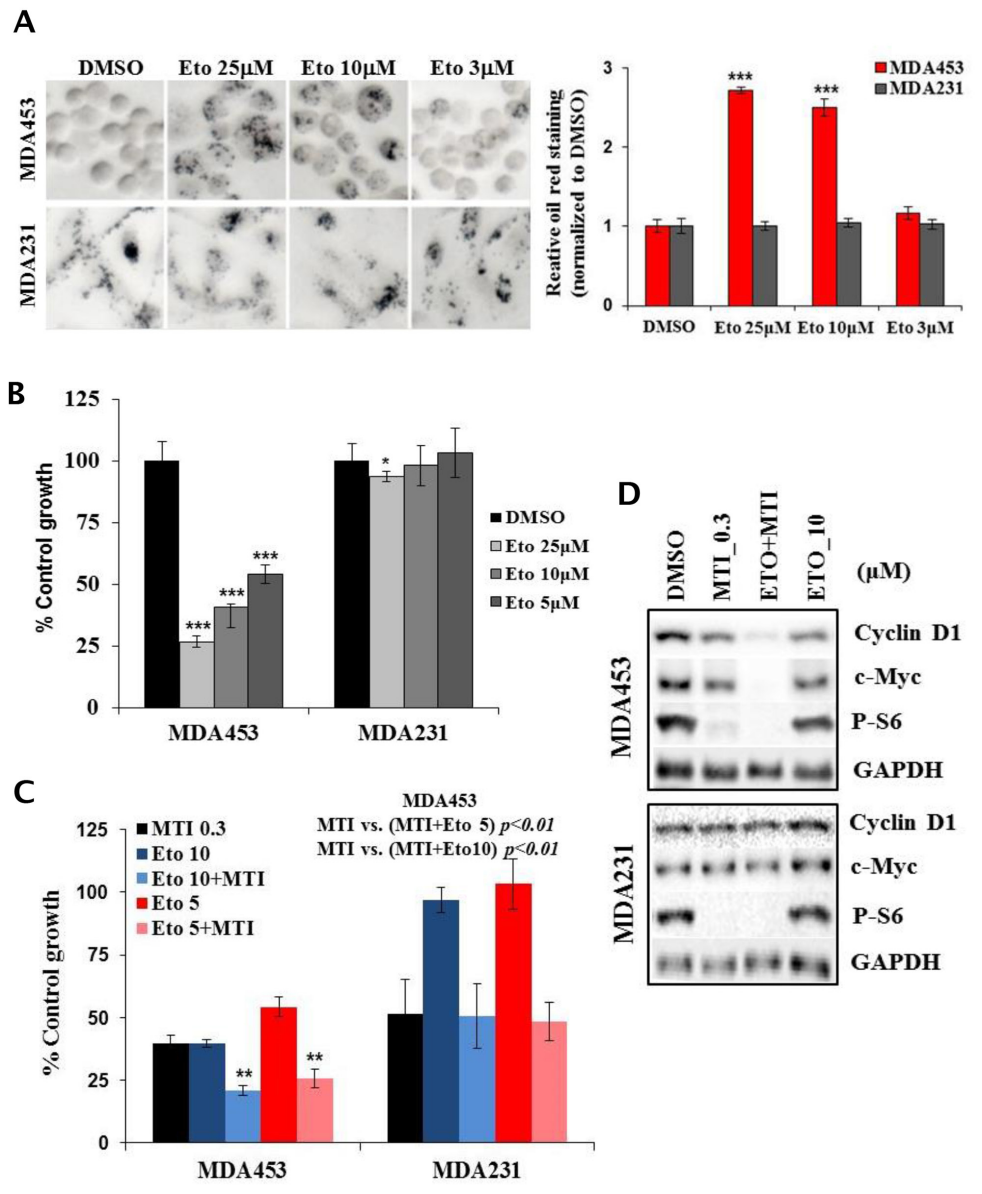
CPT1A inhibitor selectively blocks lipid catabolism and growth in MTI-31-sensitive tumor cells **A.** MDA-MB-453 and MDA-MB-231 cells were treated with DMSO and various doses of etomoxir for 24 h and subjected to Oil red O staining. The images (left panel) were quantified and plotted (right panel). **B** and **C.** MDA-MB-453 and MDA-MB-231 cells were treated with the indicated doses of etomoxir alone or in combination with 0.3 μM MTI-31 for 5 days. Cell growth was measured via cell counting. Growth inhibition by the single treatment with etomoxir (B) or combination with etomoxir and MTI-31 (C) are plotted. **D.** MDA-MB-453 cells were treated as indicated for 2 days and subjected to immunoblotting. ***, P < 0.001; **, P < 0.01.

Given that mTOR positively regulates CPT1A protein level in HER2+/PIK3CAmut cells, we considered the possibility that a direct inhibitor of CPT1A enzyme may mimic similar effects in these same cells. Indeed, acute treatment with 3, 10 and 25 μM etomoxir, a CPT1A enzyme inhibitor, resulted in a dose dependent accumulation of lipid droplets in MDA-MB-453 cells but not in MDA-MB-231 cells (Figure 7A). In cells treated with etomoxir for 5 days there was a dose proportional suppression of growth in MDA-MB-453 cells (Figure 7B). Furthermore, combination treatments using non-saturating doses of etomoxir and MTI-31 or rapamycin resulted in an enhanced growth suppression in MDA-MB-453 cells, which was not readily observed in MDA-MB-231 cells (Figure 7C, Supplementary Figure S3E), correlating a nearly complete suppression of cyclin D1 and c-Myc level in MDA-MB-453 cells under the combination treatments (Figure 7D). Together, these results in Figures 5–7 support a role for mTOR in lipid synthesis and utilization particularly in the HER2/PIK3CA-hyperactive breast cancer cells, which contribute to the antitumor efficacy of mTOR-targeted therapy.

## DISCUSSION

In this report, we have characterized the target profile of a new mTOR-KI MTI-31. MTI-31 potently binds mTOR and is selective against a panel of 98 human kinases including those related PI3K family isoforms. MTI-31 inhibited mTORC1/mTORC2 signaling in several relevant tumor models with EC_50_ values ≤120 nM. Owing to its target potency and favorable pharmacokinetic properties, MTI-31 inhibited mTOR signaling function in vivo and demonstrated single agent oral antitumor efficacy in tumor models of HER2+/PIK3CAmut breast cancer, PTEN/VHL-null renal cancer and others. These properties make MTI-31 a good pharmacological tool for dissecting mechanistic role of mTOR in vitro and in vivo.

The antitumor activity of MTI-31 was dependent on driver mutation status of the tumors. A complete suppression of tumor growth and tumor regression occurred in the HER2+/PIK3CAmut MDA-MB-453 tumors, in which a significant induction of apoptosis was present. However, MTI-31 exhibited only a minimal efficacy in the HER2-/PIK3CAwt HCC1806 tumors. The relationship of mTOR-targeted efficacy with oncogenic dysregulation of mTOR signaling was further studied in a panel of breast cancer lines. MTI-31 and AZD8055 induced a substantial cell death in the HER2+/PIK3CAmut MDA-MB-453, MDA-MB-361, BT474 and T47D cells while these inhibitors were generally cytostatic in HER2-/PIK3CAwt MDA-MB-231 and HCC1806 cells. The differential efficacy response was not due to an inadequate drug exposure or lacking suppression in mTORC1/mTORC2 substrate phosphorylation; rather, it reflected MTI-31's differential suppression of cyclin D1, c-Myc and differential induction of cell death in the HER2+/PIK3CAmut versus HER2-/PIK3CAwt cells.

Our results highlighted a role for mTORC2 in survival mechanism of HER2+/PIK3CAmut cells. Unlike rapamycin, MTI-31 induced a relatively rapid and substantial apoptosis correlating a profound suppression of c-Myc, MCL-1 and induction of Bim. A recent report showed that mTORC2 is required for suppression of MCL-1 protein degradation thereby contributing to cell survival [27]. We have found that targeting of mTORC2 by MTI-31 or depletion of cellular rictor resulted in an induction of Bim in the HER2+/PIK3CAmut cells. Depletion of Bim via ShRNA significantly attenuated the MTI-31-induced apoptosis. To our knowledge, these data provided for the first time a direct link for mTORC2-dependent molecular regulation of Bim. The importance of mTORC2/AKT in survival was further reflected in the activating dephosphorylation of GSK3 in MTI-31- or AZD8055-treated cells, in which apoptosis induced by both mTOR-KIs were partially prevented by a specific GSK3 kinase inhibitor SB415286. It thus strongly suggests that the mTORC2-mediated survival mechanism plays a critical role in HER2+/PIK3CAmut cancer cells and contributes to the therapeutic response to mTORC1/mTORC2 inhibitors.

While mTOR is best known for promoting mRNA translation [7], its role in lipid synthesis is increasingly recognized [28, 29]. Recent studies have shown that mTORC1 stimulates lipid synthesis through sterol responsive element binding proteins (SREBP1/2) [30, 31]. We have shown that MTI-31 but not rapamycin inhibited Ser-455 phosphorylation of ACL, a critical enzyme in glucose-derived de novo lipogenesis. Compared to rapamycin, MTI-31 induced a more substantial downregulation of cellular de novo lipid synthesis and acetyl-CoA level, the process that are in part dependent on ACL. We have recently shown that ACL-phosphorylation is prevalent in invasive breast tumors and occurs in an mTORC2-dependent manner [32]. Dysregulation of mTORC2-ACL in tumorigenic mechanism is consistent with recent reports that ACL plays an important role in tumor cell growth and survival and that ACL is a potential therapeutic target [33–37].

We have uncovered a novel link among mTOR, CPT1A and lipid utilization in HER2+/PIK3CAmut cells. Analysis of TCGA and CCLE databank revealed a higher CPT1A mRNA expression in the Luminal B and/or HER2+ breast cancers. A recent report showed that the CPT1A gene is amplified and presents as a genetic driver of proliferation in Luminal breast cancer [38]. In current study, mTOR-targeted antitumor efficacy in the HER2+/PIK3CAmut cells was associated with a downregulation of CPT1A protein and accumulation of cellular lipid droplet level, likely resulting from an attenuation of lipid catabolism. This pharmacological effect appears to be mediated through targeting of mTORC1 since it was observed in both the mTOR-KI- and rapamycin-treated cells. Although we are unable to attribute definitively the downregulation of CPT1A as a sole mechanism, a CPT1A enzyme inhibitor etomoxir induced a strikingly similar and selective accumulation of lipid droplet level in HER2+/PIK3CAmut cells. The fact that the HER2+/PIK3CAmut cells exhibit a higher basal CPT1A activity and lower lipid droplet level is consistent with a heightened state of lipid utilization in these cells, which could potentially in concert with lipogenesis to facilitate tumor cell growth. This notion is supported by the finding that etomoxir inhibited growth of MDA-MB-453 cells at the doses that reduced lipid utilization. Recent reports have identified CPT1A as supporting tumor growth and survival in several cancer types including that of leukemia and lymphoma [39, 40], prostate cancer [41] and ovarian cancer [42]. Our results are consistent with these reported observations and further suggest a mechanistic role for downregulation of CPT1A and lipid catabolism in mTOR-targeted therapy.

In summary, our results with MTI-31 have explored several new mechanistic aspects relevant for therapeutic response. Given that mTORC2 is required for oncogenic mechanism of EGFR, HER2 and loss of PTEN [43–45] and mTORC2 is also activated via genetic mutations in the mTORC2 components Rictor or Sin1 in cancer patients [46, 47], successful development of new generation mTORC1/mTORC2 inhibitors will facilitate an improved strategy for targeted cancer therapy.

## MATERIALS AND METHODS

### Chemicals and shRNA

MTI-31 was synthesized in Shanghai Institute of Materia Medica, Chinese Academy of Sciences as previously described for example #44 [26]. Rapamycin, AZD8055, Lapatinib and GDC-0941 were purchased from BiochemPartner (Shanghai). SB415286 (Selleck Chemicals), Etomoxir (MedChem Express) and Oil red O (Sigma-Aldrich) were purchased. All other chemicals were purchased from Sigma-Aldrich unless otherwise specified. Inhibitors were dissolved in DMSO as 20 mM stock solution and were diluted before assays. pGIPZ- and/or pTRIPZ (inducible with doxycycline)-based lentiviral shRNA for human Bim ShRNA#3 and ShRNA#6 (V3LHS_411602, V2LHS_238924), Raptor ShRNA#2 (V3LHS_329849), Rictor ShRNA#4 (V2THS_225915) and non-targeting (NT, RHS4346) were obtained from Open Biosystems/GE Dharmacon.

### Kinase assays

mTOR binding constant (Kd) of MTI-31 and binding assays for a panel of 98 protein kinases with MTI-31 at a fixed 1000 nM were performed by KINOMEscan^™^ Profiling Service (DiscoveRx Corporation). mTOR substrate phosphorylation assay was performed using recombinant mTOR enzyme (Millipore, 14-770M), 50 nM unphosphorylated 4EBP1 and 100 μM ATP, detected using the LANCE® TR-FRET platform (PerkinElmer). IC_50_ values were generated using Graphpad PRISM 5 software.

### Cell culture and gene knockdown

Cell lines of MDA-MB-453, MDA-MB-361, MDA-MB-231, BT-474, HCC1806, 786-O and U87MG were obtained from American Type Culture Collection (ATCC). T47D was obtained from the Cell Bank of Chinese Academy of Sciences (CAS, Shanghai). Cells were cultured in a 37°C incubator with 5% CO_2_ using standard cell culture methods and reagents (Invitrogen). To create gene depletion, various ShRNA constructs were packaged in 293T cells and validated according to manufacturer's instruction. To obtain stable cell population, the infected cells were selected by a pre-determined concentration of puromycin.

### Cell lysates and immunoblotting

For standard assay of inhibitor effects on cellular biomarker expression, cells were plated at pre-determined density in 6-well or 12-well culture plates for overnight, treated with inhibitors for the indicated doses and times. Cells were lysed in NuPAGE-LDS lysis buffer (Invitrogen) and immunoblotted with antibodies including P-S6K1(T389), S6, P-4EBP1(T70), 4EBP1, P-GSK3(S21/9), GSK3, cyclin D1, Raptor, Rictor, P-ACL(S455), ACL, cleaved-PARP (Cell Signaling Technology); P-S6(S235/6), AKT(S473), AKT, Bim (Abcam-Epitomics); c-Myc (Santa Cruz); Mcl-1 (Affinity); CPT1A (Proteintech); β-actin and GAPDH (Bioworld).

### Cell proliferation, survival, cell cycle and mitochondrial membrane potential

Cell proliferations were assayed with inhibitor treatment for 3 days using MTS reagent [48]. Cell survival assays were initiated at 20-30% confluence with inhibitor treatment for 3 days. Viable cells were counted by trypan-blue exclusion method or by assay using MTS reagent. Net growth or death was assessed relative to the cell density at initiation of treatment. For cell cycle profiling, cells were treated as indicated and analyzed in a Becton Dickinson FACSCalibur flow cytometer by collecting 10000 events. Mitochondrial membrane potential was assessed using 5,5′,6,6′-tetrachloro-1,1′,3,3′-tetraethylbenzimidazole carbocyanideiodide (JC-1) (AAT Bioquest) or tetramethylrhodamine ethyl ester (TMRE, Sigma). Cells with various treatments were stained with JC-1 or TMRE in serum free medium at 37°C for 30 min, washed with PBS and monitored under fluorescent microscope or fluorescence plate reader.

### Assays of de novo lipid synthesis and acetyl-CoA

Cellular glucose-to-lipid conversion was measured as described [33]. Briefly, cells were pretreated with inhibitors for 2 h, incubated for 2 h in medium containing 2 μCi/mL D-[1-14C] glucose (PerkinElmer, NEC043X001MC). Total lipids were extracted twice with 300 μL hexane:isopropanol (3:2; vol:vol). Cell debris was carefully removed by centrifugation at 14,000 rpm for 5 min using a microcentrifuge. Radioactive lipids were dried, redissolved with 100 μL of chloroform and determined by liquid scintillation counting. A duplicate assay plate without radioactive glucose label was used to count the number of cells per treatment. Glucose to total lipid conversion rate is the radioactive counts from the extracted lipid normalized by the number of cells per hour of ^14^C-glucose incubation. For acetyl-CoA assay, cells grown in 96-well plates were washed and lysed in 40 μL/well using lysis buffer [49]. Cell lysates were transferred to V-bottom plates, cleared by centrifugation. 30 μL of the cleared cell lysates were subjected to acetyl-CoA measurement using an acetyl-CoA assay kit (Sigma, MAK-039) following manufacturer's assay manual. Duplicate assay plates were used to count the number of cells per treatment for normalization in determining cellular acetyl-CoA levels.

### Oil red O staining

After treatment with the indicated inhibitors, cells were fixed in 4% buffered neutral formalin for 15 min, washed twice with PBS, stained with Oil red O (0.5% in 100% isopropanol, diluted with dH_2_O in the ratio of 3:2) for 30 min. The cells were washed 3 times with PBS to get a clear background. Pictures were captured at 200X magnification under microscope. The dye was then eluted using isopropanol and measured by spectrophotometer at 492 nm. Duplicate cell assay plate without dye-staining was used to count the number of cells per treatment. For oil red staining of tumors, tumor tissues were rehydrated using sucrose solution, embedded in Tissue-tek OCT compound gel and frozen sectioned. The slides were stained with Oil red O solution for 15 min. and sequentially washed using 75% ethanol and water.

### In vivo studies of efficacy, biomarker, apoptosis and immunohistochemistry

Animal studies were performed under protocols approved by Institutional Animal Care and Use Committee (IACUC) of Fudan University. Balbc female nude mice bearing MDA-MB-453, HCC1806 or 786-O tumors were staged at initial tumor volume of 150-200 mm^3^ and randomized into treatment groups (n=/>8). MTI-31 (salt-form) was formulated in citrate buffer (pH 4.5). Lapatinib and Sunitinib were formulated in 0.5% hydroxypropyl methyl cellulose (HPMC)-0.2% tween-80 as homogeneous suspension. All test agents were prepared twice weekly. Mice were dosed orally once daily (qd). Tumor growth inhibition rate (TGI) was calculated: TGI=[1-(Vt-Vi)/(Vc-Vi)]×100, where Vt: tumor volume of drug treated group, Vc: tumor volume of vehicle control group, Vi: initial tumor volume at staging. For biomarker studies, frozen tumors were made lysates and immunoblotted as previously described [50]. For detecting apoptosis, tumors were excised, fixed in 4% formalin for 24 h and embedded in paraffin. Slides were processed for terminal deoxynuleotidyl transferase dUTP nick end labeling (TUNEL) using an assay kit (KeyGen Biotech, KGA7061) following vendor's instruction manual. For IHC staining, tumor slides described above were deparaffinized, rehydrated and permeablized with 1% Triton X-100. Antigens were retrieved with Tris/EDTA (pH 9.0) under microwave heating for 20 min. The slides were blocked in Tris buffered saline with 5% BSA-0.3 M glycine and reacted with anti-CPT1A (Proteintech #15184-1-AP) or anti-P-ACL S455 (Abcam #59297) and detected with peroxidase-conjugated secondary antibody (Jackson # 111-035-003). Images were acquired using Leica microscope (model DMI4000D). Staining levels were analyzed on the basis of mean optical density quantified by software image pro plus 6.0.

### Statistical analysis

All numerical data processing and statistical analysis were performed with Miscrosoft Excel Software; values were expressed as means ± SD. *P* values were calculated using unpaired two-tailed Student-t test.

## SUPPLEMENTARY MATERIAL FIGURES AND TABLE




