# Supplementary material for: Molecular regulation of apoptotic machinery and lipid metabolism by mTORC1/mTORC2 dual inhibitors in preclinical models of HER2+/PIK3CAmut breast cancer
Source: Oncotarget. 2016 Aug 22;7(41):67071–86. doi: 10.18632/oncotarget.11490 (PMC5341858; doi:10.18632/oncotarget.11490)
Supplement: Supplementary file 1 [file oncotarget-07-67071-s001.pdf]

Molecular regulation of apoptotic machinery and lipid metabolism by mTORC1/mTORC2 dual inhibitors in preclinical models of HER2+/PIK3CAmut breast cancer

SUPPLEMENTARY FIGURES AND TABLE

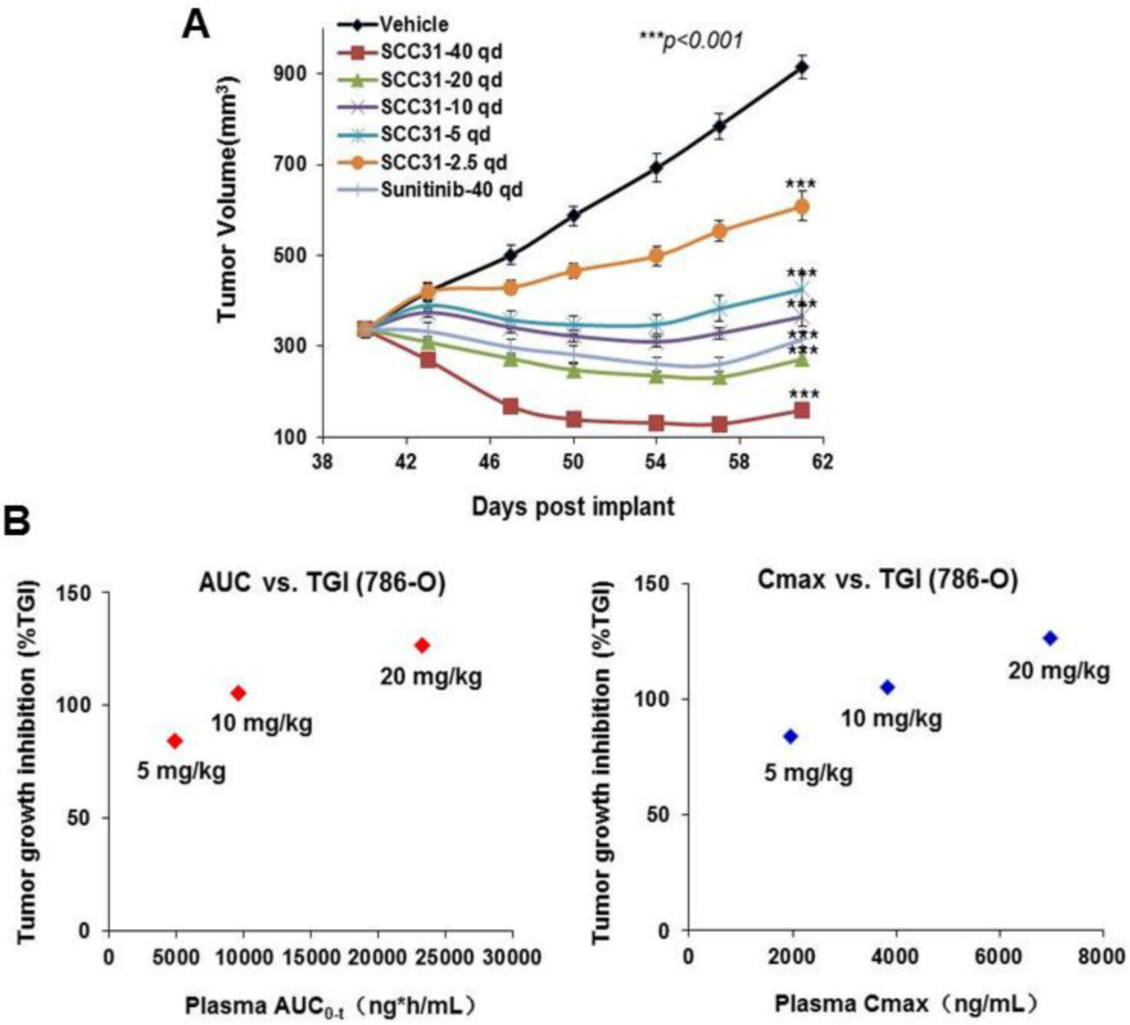

Supplementary Figure S1: In vivo efficacy response, pharmacokinetic and pharmacodynamics profile of MTI-31. **A.** Nude mice bearing 786-O tumors were treated orally with vehicle, the indicated doses of MTI-31 or a reference compound sunitinib via a once daily (qd) regimen. Tumor growth curves of a representative study are shown. **B.** Nude mice were dosed with 20, 10 and 5 mg/kg MTI-31 and analyzed for pharmacokinetics (PK). PK parameter AUC (left panel) or Cmax (right panel) versus tumor growth inhibition (TGI, mean of 2 studies) are plotted. (*Continued*)

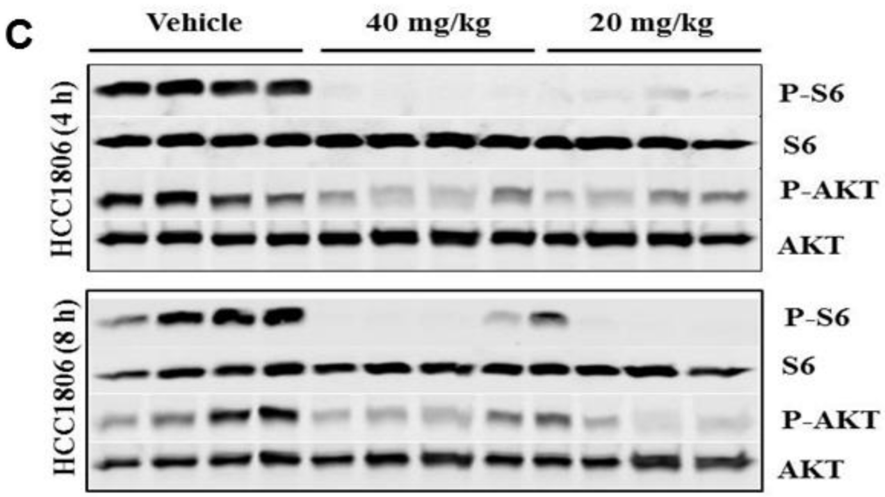

Supplementary Figure S1: (*Continued*) In vivo efficacy response, pharmacokinetic and pharmacodynamics profile of MTI-31. **C.** Nude mice bearing HCC1806 tumors were treated oral daily with 40 mg/kg or 20 mg/kg MTI-31 (Figure 2B). Tumor lysates prepared at 4 h and 8 h post the last dosing were immunoblotted as shown.

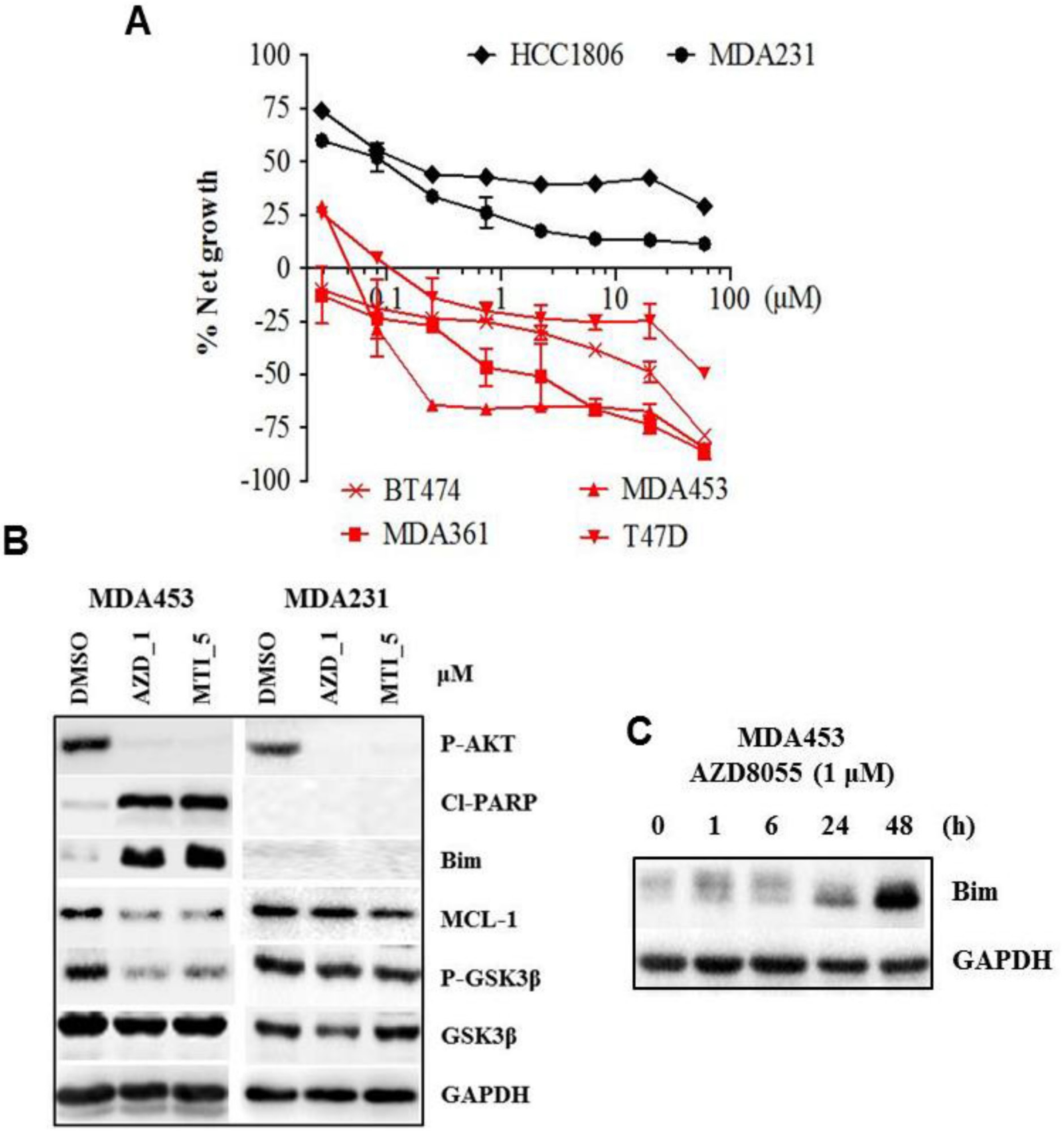

Supplementary Figure S2: AZD8055 antitumor activity correlates with cancer driver mutations. A. The indicated panel of 6 breast cancer cell lines were plated in 96-well plates and treated with various doses of AZD8055 for 3 days, analyzed for net cell growth and death as described in Methods. The cell survival dose response curves for all cell lines are plotted. B. MDA-MB-453 and MDA-MB-231 cells were treated with 1 µM AZD8055 or 5 µM MTI-31 for 48 h and subjected to immunoblotting as indicated. **C.** MDA-MB-453 cells were treated with 1 µM AZD8055 for the indicated times and subjected to immunoblotting.

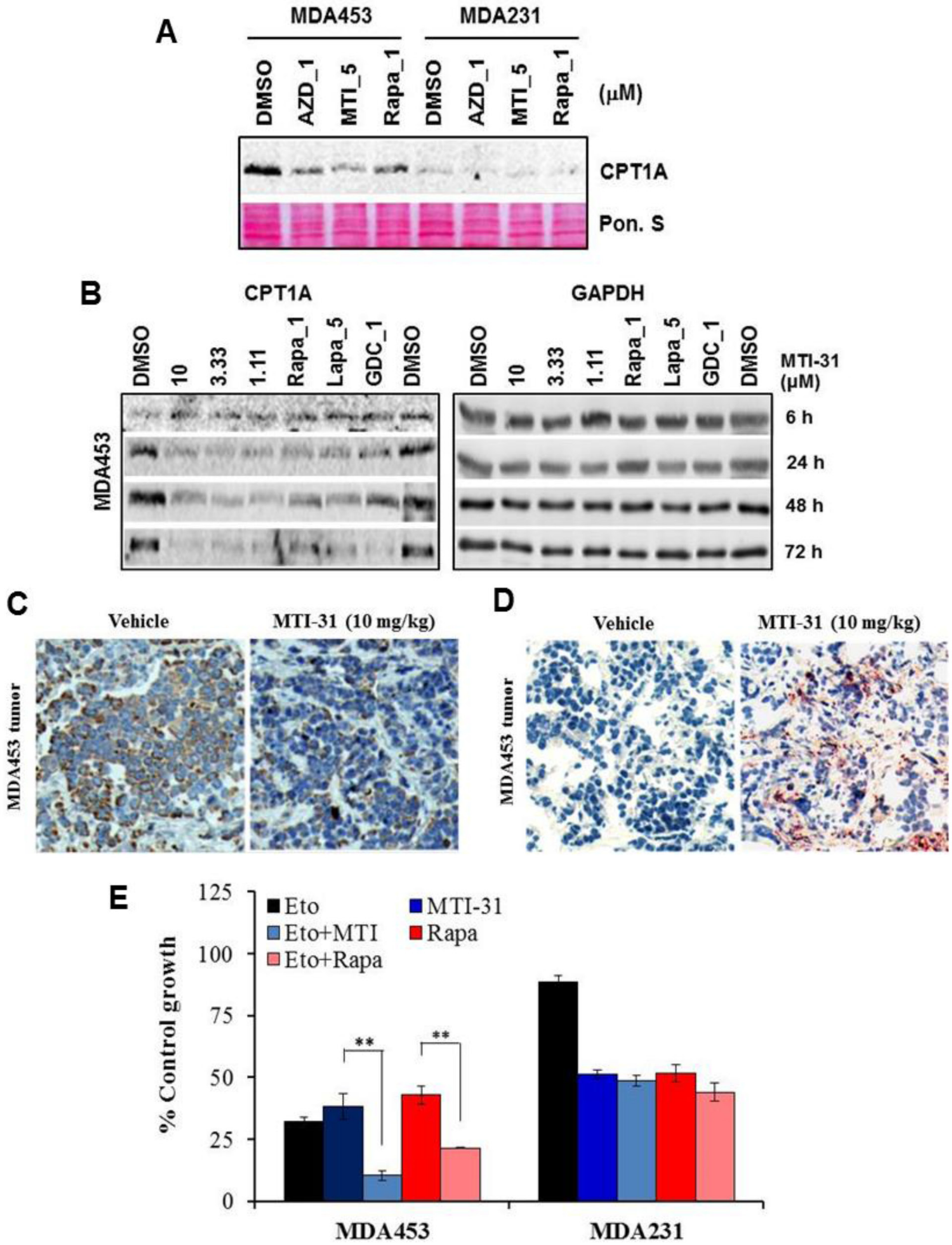

Supplementary Figure S3: MTI-31 reduces lipid catabolism in MDA-MB-453 cells. A. MDA-MB-453 and MDA-MB-231 cells were treated with 1 µM AZD8055, 5 µM MTI-31, 1 µM rapamycin for 24 h and immunoblotted. **B.** MDA-MB-453 cells were treated with various doses of MTI-31, 1 µM rapamycin, 5 µM Lapatinib, 1 µM GDC-0941 for 6, 24, 48 and 72 h. The treated cells were immunoblotted. **C** and **D.** Nude mice bearing MDA-MB-453 tumors were treated orally with MTI-31 (Figure 2A) and tumors were collected at the end of study. Tumors of vehicle- and 10 mg/kg MTI-31-treated groups were subjected to immunohistochemistry (IHC) detection with CPT1A antibody (C) and Oil red O staining (D). **E.** MDA-MB-453 and MDA-MB-231 cells were treated with 10 µM etomoxir, 0.3 µM MTI-31 or 0.1 µM rapamycin alone or in combination as indicated for 5 days. Cell growth was measured via cell counting.

Supplementary Table S1: Selectivity profile of MTI-31 against a panel of 98 kinases as assessed via KINOMEscan™ (DiscoveRx Corporation, Fremont, CA). The assays were performed with 1000 nmol/L MTI-31. Values are percent control relative to the vehicle.

See Supplementary File 1
