# Supplementary material for: Molecular regulation of apoptotic machinery and lipid metabolism by mTORC1/mTORC2 dual inhibitors in preclinical models of HER2+/PIK3CAmut breast cancer
Source: Oncotarget. 2016 Aug 22;7(41):67071–86. doi: 10.18632/oncotarget.11490 (PMC5341858; doi:10.18632/oncotarget.11490)
Supplement: Supplementary file 2 [file oncotarget-07-67071-s002.docx]

**Table S1.** Selectivity profile of MTI-31 against a panel of 98 kinases as assessed via KINOMEscan™ (DiscoveRx Corporation, Fremont, CA). The assays were performed with 1000 nmol/L MTI-31. Values are percent control relative to the vehicle.

| **Target** | **MTI-31 (1000nM)** | **Target** | **MTI-31 (1000nM)** |
| --- | --- | --- | --- |
| **Gene Symbol** | **% Control** | **Gene Symbol** | **% Control** |
| mTOR | 0.9 | AKT2 | 94 |
| ABL1-nonphosphorylated | 52 | EGFR | 94 |
| GSK3B | 56 | PAK2 | 94 |
| TYK2 (JH1domain-catalytic) | 63 | JNK2 | 96 |
| BTK | 68 | JNK3 | 96 |
| IKK-beta | 69 | ADCK3 | 97 |
| ABL1-phosphorylated | 70 | AURKA | 97 |
| MARK3 | 71 | AXL | 97 |
| ZAP70 | 72 | ERBB2 | 97 |
| MEK1 | 73 | KIT(D816V) | 97 |
| ROCK2 | 73 | PDPK1 | 97 |
| MEK2 | 75 | EPHA2 | 98 |
| ABL1 (T315I)- phosphorylated | 76 | PKAC-alpha | 98 |
| DCAMKL1 | 76 | VEGFR2 | 98 |
| ABL1 (E255K)- phosphorylated | 78 | CDK3 | 99 |
| ALK | 78 | CDK7 | 99 |
| PLK1 | 78 | CSF1R | 99 |
| RSK2 (Kin.Dom.1-N- terminal) | 78 | AKT1 | 100 |
| AURKB | 80 | CDK11 | 100 |
| BRAF | 80 | CSNK1G2 | 100 |
| CSNK1D | 80 | DYRK1B | 100 |
| RIOK2 | 80 | EGFR (L858R) | 100 |
| ULK2 | 80 | ERBB4 | 100 |
| JNK1 | 81 | ERK1 | 100 |
| IKK-alpha | 82 | FGFR2 | 100 |
| JAK3 (JH1domain-catalytic) | 82 | FLT3 | 100 |
| RAF1 | 82 | IGF1R | 100 |
| CHEK1 | 83 | INSR | 100 |
| CDK2 | 84 | KIT | 100 |
| TRKA | 84 | KIT (V559D, T670I) | 100 |
| YANK3 | 85 | LKB1 | 100 |
| ACVR1B | 86 | MAP3K4 | 100 |
| MAPKAPK2 | 87 | MET | 100 |
| PCTK1 | 87 | MKNK2 | 100 |
| PLK4 | 87 | MLK1 | 100 |
| BMPR2 | 88 | p38-alpha | 100 |
| JAK2 (JH1domain-catalytic) | 88 | p38-beta | 100 |
| FAK | 89 | PAK1 | 100 |
| SNARK | 89 | PAK4 | 100 |
| CDK9 | 90 | PDGFRB | 100 |
| MKNK1 | 90 | PIK3C2B | 100 |
| SRPK3 | 90 | PIK3CA | 100 |
| PRKCE | 91 | PIK3CG | 100 |
| FGFR3 | 92 | PIM1 | 100 |
| PIM2 | 92 | PIM3 | 100 |
| PLK3 | 92 | RET | 100 |
| BRAF (V600E) | 93 | SRC | 100 |
| PDGFRA | 93 | TIE2 | 100 |
| TGFBR1 | 93 | TSSK1B | 100 |
